# Efficient production of heat-stable antifungal factor through integrating statistical optimization with a two-stage temperature control strategy in *Lysobacter enzymogenes* OH11

**DOI:** 10.1186/s12896-018-0478-2

**Published:** 2018-10-24

**Authors:** Bao Tang, Cheng Sun, Yancun Zhao, Huiyong Xu, Gaoge Xu, Fengquan Liu

**Affiliations:** 1 Institute of Plant Protection, Jiangsu Academy of Agricultural Sciences, Nanjing, 210014 China; 2 School of Resources and Environmental engineering, Yangzhou Polytechnic College, Yangzhou, 225009 China

**Keywords:** Heat-stable antifungal factor, *Lysobacter enzymogenes* OH11, Biopesticides, Two-stage temperature control strategy

## Abstract

**Background:**

Heat-stable antifungal factor (HSAF) is a newly identified broad-spectrum antifungal antibiotic from the biocontrol agent *Lysobacter enzymogenes* and is regarded as a potential biological pesticide, due to its novel mode of action. However, the production level of HSAF is quite low, and little research has reported on the fermentation process involved, representing huge obstacles for large-scale industrial production.

**Results:**

Medium capacity, culture temperature, and fermentation time were identified as the most significant factors affecting the production of HSAF and employed for further optimization through statistical methods. Based on the analysis of kinetic parameters at different temperatures, a novel two-stage temperature control strategy was developed to improve HSAF production, in which the temperature was increased to 32 °C during the first 12 h and then switched to 26 °C until the end of fermentation. Using this strategy, the maximum HSAF production reached 440.26 ± 16.14 mg L^− 1^, increased by 9.93% than that of the best results from single-temperature fermentation. Moreover, the fermentation time was shortened from 58 h to 54 h, resulting in the enhancement of HSAF productivity (17.95%) and yield (9.93%).

**Conclusions:**

This study provides a simple and efficient method for producing HSAF that could be feasibly applied to the industrial-scale production of HSAF.

**Electronic supplementary material:**

The online version of this article (10.1186/s12896-018-0478-2) contains supplementary material, which is available to authorized users.

## Background

Heat-stable antifungal factor (HSAF) is mainly produced by *Lysobacter enzymogenes*, a gram-negative bacterium used in biological control in agriculture. Belonging to the polycyclic tetramate macrolactams (PTMs), HSAF exhibits a potent and broad antagonistic activity against fungi and oomycetes. More importantly, its mode of action against pathogenic fungi is novel and different from those of the commercial fungicides that have been reported. It was proved that HSAF inhibits the polarized growth of filamentous fungi by disrupting the biosynthesis of sphingolipids, which differs between fungal and mammalian cells [1]. Therefore, HSAF is believed to be safe for the environment and humans and has the possibility of being developed as a “green pesticide” in the future.

As a novel antifungal active substance, there has been an increasing progress in the research of HSAF as an alternative biopesticide around the world. The key genes involved in the biosynthesis of HSAF were identified as *pks/nrps* in *L. enzymogenes* C3 [2]. And the synthesis mechanism was revealed as follows: two polyketide precursors were synthesized synchronously by a single polyketide synthase (PKS), and then assembled with ornithine to form a macrocyclic lactam via cyclization or cycloaddition reactions by a nonribosomal peptide synthetase (NRPS) [3, 4]. Other studies also indicated that the biosynthesis of HSAF was regulated by a large number of key factors that could be divided into two main categories: positive regulatory factors (*Le*DSF [5], Clp [6], and Lsp [7]) and negative regulatory factors (LesR [8], PilR [9], and LetR [10]). However, little work has been conducted to study the production of HSAF through fermentation, probably because the biosynthesis of HSAF requires harsh conditions in the culture environment [2]. Recent advances have started to focus on this issue, and HSAF production by *L. enzymogenes* OH11 was increased with a screened medium to 356.34 mg L^− 1^, which is approximately 12-fold higher than that of a conventional medium (10%TSB, 29.34 mg L^− 1^) [11]. Nevertheless, this production level is still lower than that required for large-scale industrial production, and the effects of fermentation parameters on the production of HSAF have not been studied. Consequently, it is necessary to investigate fermentation conditions to maximize HSAF production.

Many studies have shown that the optimum fermentation conditions for cell growth and metabolite formation were usually quite different during fermentation, which had a serious impact on the accumulation and production of secondary metabolites. A two-stage control strategy has been adopted in most microbial fermentations to solve this problem. The first stage can be regarded as a cell growth stage in which cell density increased rapidly, but the cells accumulated a low content of metabolites. The second stage can be considered as a product accumulation stage in which cell numbers increased little but secondary metabolite levels in the cells, cell body weight and cell size all increased, leading to a high accumulation of product in the fermentation broth [12]. For example, the highest arachidonic acid production of 8.12 g L^− 1^ was achieved with a two-stage pH control strategy, which was higher than the best results achieved at a constant pH (7.43 g L^− 1^) [13]. Through application of a two-stage temperature control strategy to 1,3-propanediol production, the fermentation time was shortened from 10 h to 9.2 h, resulting in an increase in 1,3-propanediol productivity of approximately 11% [14]. A relatively high acetoin concentration (44.9 g L^− 1^) and high acetoin productivity (1.73 g L^− 1^ h^− 1^) were achieved by developing a two-stage agitation speed control strategy [15]. A two-stage oxygen supply control strategy was proposed for the fermentation of docosahexaenoic acid (DHA), and the DHA content and productivity reached 17.7 g L^− 1^ and 111 mg L^− 1^ h^− 1^, respectively, which were 63.88% and 32.14% higher than the best results from experiments in which a constant K_L_a was maintained [16]. These studies have all proved that two-stage fermentation strategies not only increase the output of target products but also improve production efficiency.

In this study, various culture conditions affecting the production of HSAF were systematically investigated for the first time, and the significant parameters were screened and further optimized through statistical analysis. Subsequently, a two-stage temperature control strategy was proposed to improve the production and productivity of HSAF based on a kinetic analysis of batch processes. The resulting control strategy may provide guidance for industrial-scale production of HSAF.

### Materials

The strain for HSAF production used in this study was *L. enzymogenes* OH11, originally isolated from soil in which vegetables and deposited afterward in the China General Microbiological Culture Collection Center (No. 1978) [11].

The seed medium was Luria-Bertani contained the following (in g L^− 1^): tryptone, 10; yeast extract, 5; NaCl, 10. The fermentation medium was comprised the following (in g L^− 1^): soybean flour, 8.00; glucose, 7.89; CaCl_2_, 0.72. The initial pH was adjusted to 7.0.

## Methods

### Cultivation conditions

A loop of *L. enzymogenes* OH11 was first inoculated into 100 mL of seed medium in 500 mL flasks, and then aerobically incubated at 28 °C for 12 h with shaking at 180 rpm. The seed culture was transferred to a 500 mL flask containing the fermentation medium. The values of culture conditions were varied to study the effects of different fermentation parameters on HSAF production based on the experimental design.

Then, the HSAF fermentation was scaled up to a 50 L fermentor (GRJ-50D, Zhengjiang, China) containing 30 L of medium. The aeration rate was maintained at 1.2 vvm, and the agitation speed was controlled at 100 rpm. Other fermentation parameters were the same as in the shake flask under the optimized conditions or the two-stage temperature conditions.

All the experiments were carried out in triplicate, and samples were taken regularly for analyses of the studied parameters.

### Design of experiments and statistical analysis

#### Plackett-Burman design (PBD)

PBD is an effective technique for rapidly screening multiple factors to find the most significant factors among a large number of fermentation parameters [17, 18]. Herein, six factors (independent variables), including inoculation amount (X_1_), medium capacity (X_2_), initial pH (X_3_), culture temperature (X_4_), rotation speed (X_5_), and fermentation time (X_6_), were considered to identify potential factors affecting the production of HSAF (response). Each independent variable was set at two levels: a high (+ 1) and a low (− 1) level. As illustrated in Additional file 1: Table S1, 12 trials were carried out to evaluate the effects of the six experimental parameters, and the determination of HSAF production was reported as the average of three trials.

The main effect of each variable was determined with the fitted first-order model as follows:

$$ Y=\beta 0+\Sigma \beta iXi\ \left(i=1,2,\cdots \mathrm{k}\right) $$

where Y is the effect estimate; β_0_ is the model intercept; β_1_ is the linear coefficient; and X_i_ is the coded independent factor. Model equation quality was determined as the coefficient of R^2^, and its statistical significance was determined from the F-test. Design-Expert software (State-Ease, Inc., Minneapolis, USA, trial version 8.0.6.1) was used to generate and analyse the experimental design of PBD.

#### Path of steepest ascent

The factors screened via the PBD method were further optimized by the path of steepest ascent, i.e., along the direction of the maximum increase in the response. The direction is parallel to the normal to the fitted response surface, and the lengths of the steps along the path are proportional to the regression coefficient, β_i_. The results of the tests are shown in Table 2.

#### Box-Behnken design (BBD)

After the selection of three significant factors using PBD, a BBD of response surface methodology (RSM) was employed to further optimize the three most significant factors (X_2_, X_4,_ and X_6_) for enhancing the production of HSAF. The independent factors were investigated at three different levels of − 1 (lower), 0 (middle) and + 1 (higher), and a total of 17 experiments were formulated, as shown in Additional file 1: Table S2. The experimental results were fitted with a second-order polynomial equation, and a multiple regression of the data was carried out to obtain an empirical model related to the most significant factors. The 3D contour graphs were displayed to obtain information about significant effects and interactions between the selected factors with positive influence on the HSAF production. The BBD was generated by Design-Expert software. All experiments were carried out in 500-mL Erlenmeyer flasks as described earlier and repeated in triplicate. To check the validity of the quadratic model, three experiments were performed with the predicted optimal parameters, and HSAF production was estimated and compared with its predicted values.

### HSAF extraction from fermentation and quantitative determination

Three-millilitre aliquots of fermentation samples were withdrawn from the flasks and adjusted to pH 2.5 with HCl. Ethyl acetate was added to the acidified broth in a 1:1 proportion, together with 0.3 g of CaCl_2_, and the mixture was shaken in a vortexer at 2000 rpm for 1 min. After centrifugation, 1 mL of the solvent layer containing HSAF was separated and ventilated to dryness in a fume hood. The HSAF crude extract was redissolved in 1 mL of methanol and used for high-performance liquid chromatography (HPLC) analysis using an InterSustainSwift C18 column (5 μm, 250 × 4.6 mm) with detection at 318 nm. Pure water and acetonitrile containing 0.04% (*v*/v) TFA were used as the A and B mobile phases, respectively. The gradient program used a flow rate of 1.0 mL min^− 1^. The compound with a retention time of 20.59 min was identified as HSAF. Finally, the production of HSAF (mg L^− 1^) was calculated from the standard curve made by the purified HSAF concentration and the absorption peak area.

### Analytical methods

Aliquots of 10 mL of broth were withdrawn from the flasks for analysis at regular intervals, and residual soybean flour was removed through 1 min of natural sedimentation. Dry cell weight (DCW) was determined from supernatants that had been harvested by centrifugation at 10,000×g for 20 min; the resulting pellets were then washed with distilled water and dried at 80 °C to constant weigh. In addition, the glucose remaining in the supernatant was enzymatically quantified using a biosensor (SBA-40C, Shangdong Academy of Sciences, China). All experiments were performed in triplicate, and the results were expressed as the mean ± standard deviation.

## Results

### Screening of significant factors affecting HSAF production using PBD

To evaluate which physical parameters exert a significant effect on the production of HSAF by *L. enzymogenes* OH11, Plackett-Burman experiments were executed, and the results are presented in Additional file 1: Table S1. The data were analysed by Design-Expert, resulting in the fitting of the following first-order model to the experimental HSAF production: Y = 271.71–4.20X_1_ + 14.19X_2_ + 4.93X_3_ + 31.31X_4_–0.71X_5_ + 36.41X_6_. The coefficient of each variable in the formula indicates that variable’s degree of influence on HSAF production, and a + or – sign represents the positive or negative influence. A summary of the analysis of variance (ANOVA) for the selected quadratic model is shown in Table 1. The fitted model resulted in an R^2^ (coefficient of determination) value of 0.9453, which indicated that 94.53% of the variability in the response could be explained by the model. *P* values were calculated to identify the main effects of each factor, and values less than 0.05 denoted that the factor significantly affected the response [19]. X_2_, X_4_, and X_6_ were clearly the investigated factors that were most significant for HSAF production, and their variation was included in the next stage of optimization. In contrast, X_1_, X_3_, and X_5_ had non-significant effects on HSAF production, and their values were fixed at 2.5%, 7.0, and 200 rpm, respectively, in the subsequent analyses.

**Table 1:** Statistical analysis of variables

Factors	Variables	Low level (− 1)	High level (+ 1)	Coefficient	SS	F-Value	*P*-value
Inoculation amount (%)	X_1_	2	2.5	−4.20	211.51	0.60	0.4746
Medium capacity (%)	X_2_	16	20	14.19	2415.71	6.82	0.0476
Initial pH	X_3_	5.5	7.0	4.93	292.25	0.83	0.4054
Culture temperature (°C)	X_4_	22	28	31.31	11,760.04	33.20	0.0022
Rotation speed (r min^−1^)	X_5_	150	200	−0.71	6.08	0.017	0.9009
Fermentation time (h)	X_6_	36	48	36.41	15,909.71	44.92	0.0011

R^2^ = 0.9453, R^2^(Adj) = 0.8796; a Significant at 95% confidence degree (P<0.05)

### The path of steepest ascent

Based on the above results, medium capacity, culture temperature, and fermentation time were the major factors influencing HSAF production. Furthermore, the first-order model equation showed that the coefficients of X_2_, X_4_, and X_6_ were 14.19, 31.31, and 36.41, respectively, and that their ratio was approximately 2:4:5. The path of steepest ascent was then employed, and parameter values moved along the path in which medium capacity, culture temperature, and fermentation time were increased to determine the proper direction for optimization. As shown in Table 2, the highest HSAF production of 376.08 ± 15.43 mg L^− 1^ was achieved when the values of the significant factors were as follows: medium capacity of 20%, culture temperature of 28 °C, and fermentation time of 54 h, which meant that this point was near the maximum HSAF production response, and it was therefore selected as a starting point for further optimization.

**Table 2:** Design and results of the path of steepest ascent experiments

Run	Medium capacity X_2_ (%)	Culture temperature X_4_ (°C)	Fermentation time X_6_ (h)	HSAF production (mg L^−1^)
1	16%	20	44	238.33 ± 14.65
2	18%	24	49	286.47 ± 13.85
3	20%	28	54	376.08 ± 15.43
4	22%	32	59	237.48 ± 14.72
5	24%	36	64	28.38 ± 14.32

### Optimization of significant variables for HSAF production by RSM

The optimum values of the three significant factors were determined by employing the RSM using BBD, and the experimental results are shown in Additional file 1: Table S2. Applying multiple regression analysis to the experimental data generated the following second-order polynomial equation to describe HSAF production: Y = 374.53 + 12.77X_2_–41.84X_4_ + 24.32X_6_–15.65X_2_X_4_ + 8.17X_2_X_6_–12.55X_4_X_6_–20.07X_2_^2^–48.42X_4_^2^–27.97X_6_^2^; where Y is the predicted HSAF production (mg L^− 1^), and X_2_, X_4_, and X_6_ are the coded values of the three significant factors. ANOVA was executed to check the adequacy of the fitted equation, and its results are presented in Additional file 1: Table S3. Generally, a regression model with an R^2^ value above 0.9 is generally considered to show a very high correlation [20]. Here, the R^2^ value was 0.9918, indicating that this model can explain 99.18% of HSAF production in response to the variation of the three most significant parameters. Consequently, it is reasonable to utilize the regression model to predict HSAF production within the range of the variables studied. “Probe > F” is usually utilized to determine the significance of variables and reflects the strength of independent variables [21]. Smaller values indicate that the corresponding variable is more significant. The ANOVA indicated that the model terms of X_2_, X_4_, X_6_, X_2_X_4_, X_4_X_6_, X_2_^2^, X_4_^2^, and X_6_^2^ were highly significant for HSAF production, since each “Probe > F” value was less than 0.01. The interactive effects represented by X_2_X_6_ were significant. The interdependence between X_2_, X_4_, and X_6_ was predicted within their experimental ranges based on the three-dimensional response surface curves. Each response surface for HSAF production was convex, which suggested that the optimal conditions were well-defined and that an optimal value existed for each variable. Whether the interaction between the significant factors was significant to the response was indicated by the shape of the corresponding contour plot. As illustrated in Additional file 1: Figure S1A, B, and C, each contour map exhibited an elliptical shape, indicating that the interactions between X_2_, X_4_, and X_6_ were very significant, which was consistent with the ANOVA results. According to the above analysis, the predicted maximum production of HSAF was 400.55 mg L^− 1^, which would occur when these variables were at their optimal values of 22.11%, 26.10 °C, and 58.07 h, respectively, while the other variables were kept at zero levels. The predicted results were verified by performing the experiments in triplicate under conditions of approximately 22%, 26 °C, and 58 h, respectively. The observed experimental production was 400.49 ± 16.41 mg L^− 1^, in agreement with the model prediction, showing the accuracy of the experiments.

### Kinetic characteristics of HSAF fermentation at different temperatures

**Fig. 1:**
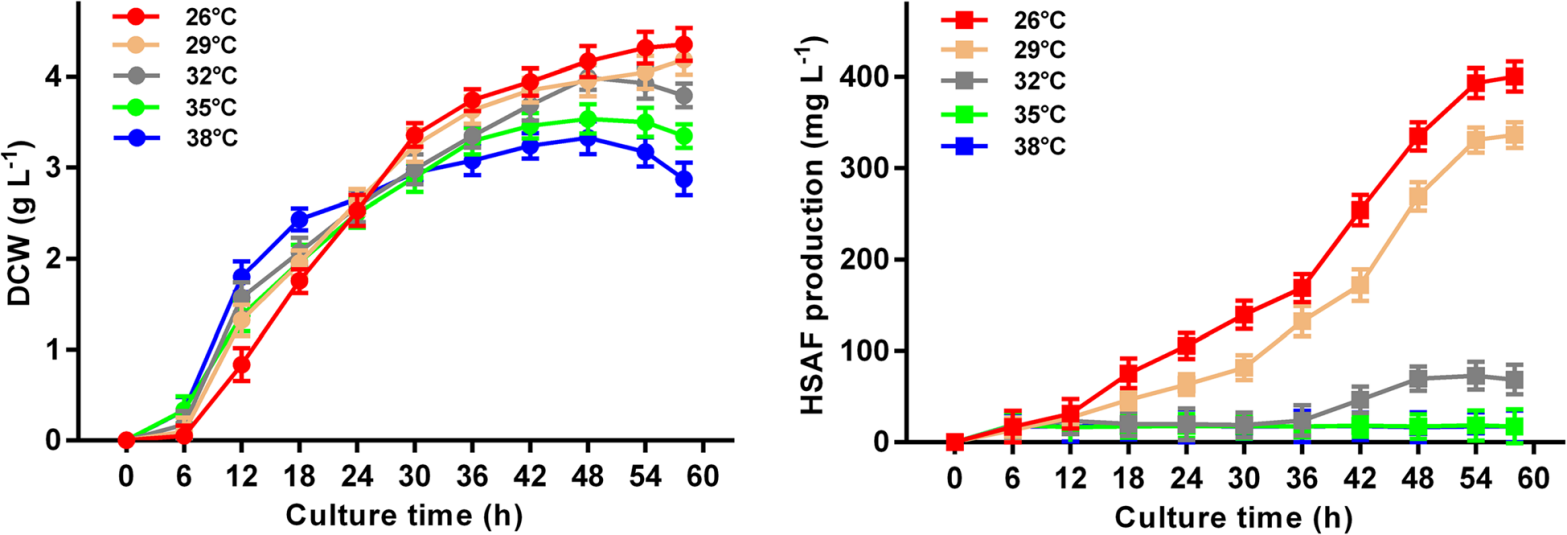
Effects of different temperatures on microbial growth (**a**) and HSAF production (**b**)

As presented in Fig. 1, the effects of various incubation temperatures (26 °C, 29 °C, 32 °C, 35 °C, and 38 °C) on microbial growth and HSAF production were examined. In the first 24 h of fermentation, *L. enzymogenes* OH11 grew faster when the fermentation temperature was increased from 26 °C to 38 °C. Subsequently, an increase in temperature led to a decrease in biomass accumulation, especially at 38 °C in late fermentation (Fig. 1a). This behaviour was most likely due to the secretion of a variety of extracellular enzymes by *L. enzymogenes* OH11 and led to a dramatic decrease in cell biomass at high temperature [22]. At the end of fermentation, the highest DCW of 4.35 ± 0.32 g L^− 1^ was obtained at 26 °C, while the lowest biomass was only 2.87 ± 0.26 g L^− 1^ at 38 °C. In a word, high temperatures can stimulate rapid growth of cells in the early stage of fermentation, while low temperatures are more conducive to subsequent biomass accumulation.

The synthesis of HSAF occurred after the growth of cells and was partially coupled to growth, similar to other secondary metabolites (Fig. 1b) [23]. The production of HSAF began to show considerable variation beginning at 12 h at different fermentation temperatures. When the culture temperature exceeded 32 °C, the biosynthesis of HSAF was essentially inhibited. This phenomenon suggests that HSAF synthase or a regulatory enzyme is probably a low-temperature-activated enzyme and gets to be inactivated at high temperature to lose its catalytic activity. By comparison, low cultivation temperatures generally favoured the production of HSAF, with the maximum titre of HSAF (400.49 ± 16.41 mg L^− 1^) being reached at 26 °C. When the temperature was lower (23 °C), HSAF production decreased markedly (data not shown). The temperature of 23 °C might be more suitable for HSAF synthesis than higher temperatures, but the lower cell growth at this temperature limits HSAF accumulation.

### Development of a two-stage temperature control strategy for HSAF fermentation

Based on the effect of culture temperature on HSAF fermentation, it was considered favourable to adopt a two-stage temperature control strategy, instead of maintaining a single constant temperature. Under this strategy, the temperature was kept at 29 °C, 32 °C, 35 °C, and 38 °C for the first 24 h, to accelerate the adaptation period, and then switched to 26 °C, to maintain high HSAF accumulation in later cultivation. The design and results of the two-stage temperature controlling experiments are listed in Table 3. The switching time and the range of temperature increase had strong effects on the fermentation of HSAF, although the experimental results were not all satisfactory. With a proper increase in temperature (to below 35 °C) over a short fermentation time, both DCW and HSAF titre were increased. Under these conditions, the maximum production of HSAF was 440.26 ± 16.14 mg L^− 1^ (under Scheme 6), which was 9.93% higher than that of fermentation with a single constant temperature (400.49 ± 16.41 mg L^− 1^). When the pre-fermentation temperature exceeded 35 °C, regardless of the switching time, DCW and HSAF production decreased significantly.

**Table 3:** Experimental design of two-stage temperature control

Scheme	Stage Ι	Stage II	DCW (g L^−1^)	HSAF production (mg L^− 1^)
Control	0–6 h:26 °C	6–58 h:26 °C	4.35 ± 0.32	400.49 ± 16.41
1	0–6 h:29 °C	6–58 h:26 °C	4.28 ± 0.28	403.45 ± 15.24
2	0–6 h:32 °C	6–58 h:26 °C	4.42 ± 0.24	407.43 ± 13.24
3	0–6 h:35 °C	6–58 h:26 °C	4.15 ± 0.27	376.52 ± 14.74
4	0–6 h:38 °C	6–58 h:26 °C	3.83 ± 0.24	312.21 ± 14.21
5	0–12 h:29 °C	12–58 h:26 °C	4.53 ± 0.26	412.99 ± 16.41
6	0–12 h:32 °C	12–58 h:26 °C	4.43 ± 0.27	440.26 ± 16.14
7	0–12 h:35 °C	12–58 h:26 °C	4.18 ± 0.28	252.03 ± 15.20
8	0–12 h:38 °C	12–58 h:26 °C	3.20 ± 0.24	225.34 ± 13.27
9	0–18 h:29 °C	18–58 h:26 °C	3.62 ± 0.27	266.00 ± 14.65
10	0–18 h:32 °C	18–58 h:26 °C	4.24 ± 0.25	191.07 ± 14.45
11	0–18 h:35 °C	18–58 h:26 °C	3.39 ± 0.22	146.88 ± 13.24
12	0–18 h:38 °C	18–58 h:26 °C	2.33 ± 0.20	164.30 ± 13.18

Scheme 6 was also carried out in the 50 L fermentor, and the final production of HSAF was 296.45 ± 13.22 mg L^− 1^, which was 26.40% higher than under constant-temperature fermentation (26 °C) (234.53 ± 11.84 mg L^− 1^). Therefore, the two-stage temperature control strategy was also applicable to HSAF fermentation at the fermentor level. However, the overall level of HSAF in the fermentor was lower than that in the shake flask, probably because the shear force of mechanical agitation exerted damage on cell growth, resulting in a low DCW of 3.23 ± 0.22 g L^− 1^. Therefore, further work should focus on parameter optimization at the fermentor level.

The proposed two-stage temperature control strategy was clearly successful at increasing HSAF production when the culture temperature was controlled at 32 °C in the first 12 h to promote cell growth and then switched to 26 °C to stimulate the biosynthesis of HSAF.

### Comparison of kinetic parameters in different temperature control modes for HSAF fermentation

The kinetic parameters of HSAF fermentation under two-stage temperature control were analysed and compared with those of constant-temperature fermentation (26 °C) to better understand the characteristics of this process.

**Fig. 2:**
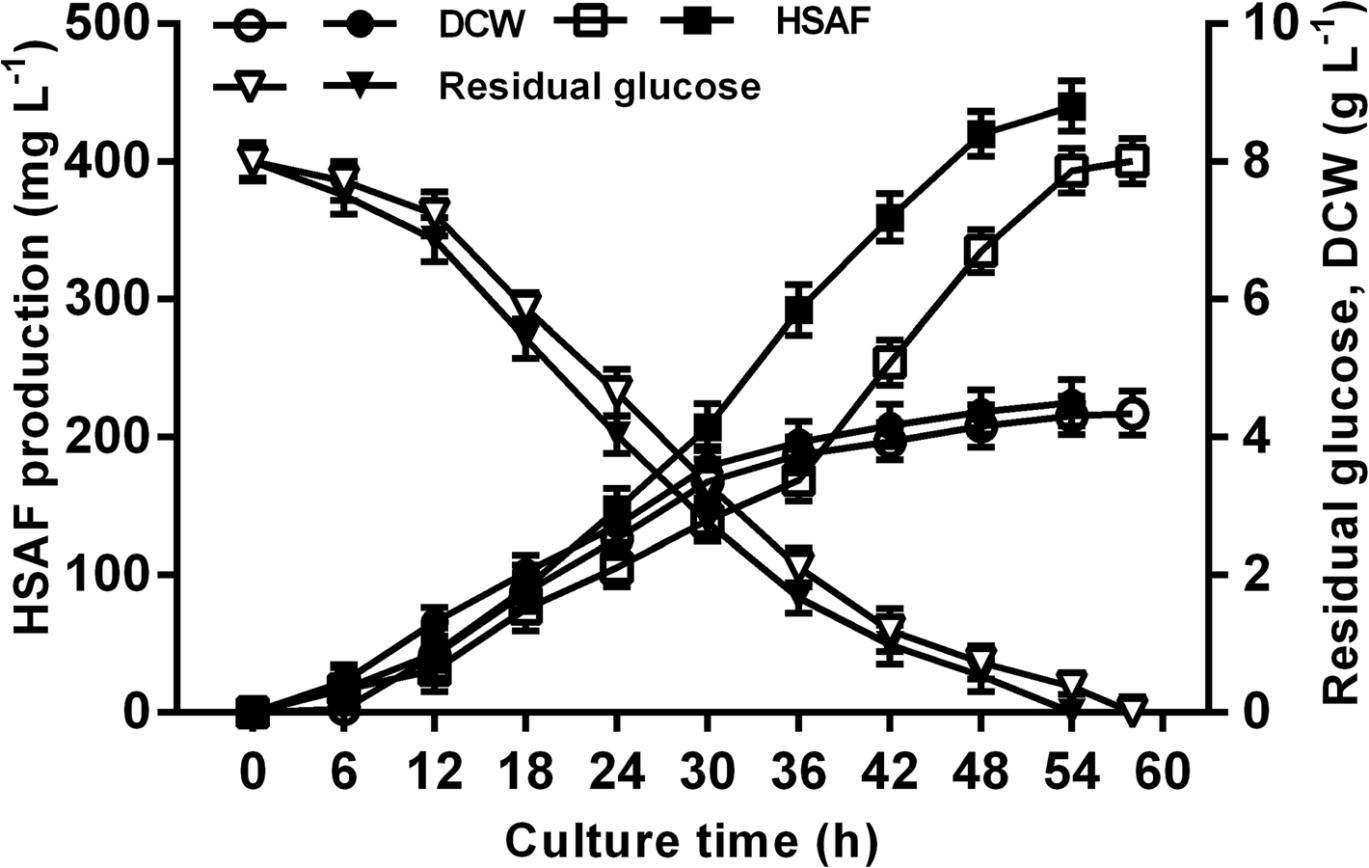
Time-course of HSAF batch fermentation by *L. enzymogenes* OH11 under different temperature control modes. The filled symbols represent HSAF fermentation under the two-stage temperature control strategy: HSAF (■), DCW (●), residual glucose (▼). The empty symbols represent HSAF fermentation under the constant-temperature strategy: HSAF (□), DCW (○), residual glucose (▽)

As shown in Fig. 2, the residual glucose content in the two-stage fermentation broth was always lower than that of the constant-temperature fermentation broth, indicating that glucose consumption was faster under two-stage temperature regulation. Thus, the fermentation period was shortened from 58 h to 54 h with two-stage temperature control, considering the point at which glucose was no longer consumed as the end of fermentation. Under a higher temperatures at the early stage of fermentation, the biomass went through the lag phase faster and acquired its maximum DCW of 4.50 ± 0.34 g L^− 1^, with a growth rate of 0.083 ± 0.006 g L^− 1^ h^− 1^, while at constant temperature, these values were 4.35 ± 0.32 g L^− 1^ and 0.075 ± 0.006 g L^− 1^ h^− 1^, respectively (Table 4). Regarding the biosynthesis of HSAF, a clearly elevated HSAF titre was generated as a result of using two phases of temperature control (Fig. 2). In particular, the fermentation efficiency of HSAF was enhanced significantly, and the HSAF productivity reached 8.15 ± 0.30 g L^− 1^ h^− 1^, increased by 17.95% than that of constant-temperature processes (6.91 ± 0.28 g L^− 1^ h^− 1^). In addition, the two-stage temperature control strategy achieved a final HSAF yield from glucose of 55.80 ± 2.05 mg g^− 1^, which was 9.93% higher than the best result from single temperature fermentations.

**Table 4:** Summary of fermentation parameters under different temperature control strategies

Temperature control modes	Fermentation time ^a^ (h)	DCW (g L^− 1^)	Growth rate (g L^− 1^ h^− 1^)	HSAF		
				production (mg L^− 1^)	productivity (mg L^−1^ h^− 1^)	yield ^b^ (mg g^− 1^)
Constant temperature	58	4.35 ± 0.32	0.075 ± 0.006	400.49 ± 16.41	6.91 ± 0.28	50.76 ± 2.08
Two-stage temperature	54	4.50 ± 0.34	0.083 ± 0.006	440.26 ± 16.14	8.15 ± 0.30	55.80 ± 2.05

^a^Fermentation time was defined as the time when the glucose was no longer consumed;

^b^HSAF yield was expressed as mg HSAF g^− 1^ glucose utilized

## Discussion

Since *L. enzymogenes* is not commonly used in the fermentation industry, its optimum culture conditions, including pH, temperature, rotation speed, and fermentation time are not well described. Herein, using RSM with an appropriate design provided significant information about the combinations of fermentation parameters that can be applied to improve HSAF production by *L. enzymogenes* OH11. Moreover, medium capacity, culture temperature, and fermentation time were shown to significantly influence HSAF production, and their levels should be strictly controlled during the fermentation process.

However, the optimum temperature was determined to be only 26 °C as the characteristics of HSAF biosynthesis by *L. enzymogenes*. Studies have shown that low-temperature fermentation results in slower cell growth, lower product synthesis, and excessive energy consumption [24]. Through analysis of the kinetic parameters of HSAF fermentation at different temperatures, it was found that the culture temperature clearly played a vital role in cell growth and HSAF production. Although the maximum cell biomass and HSAF production could be acquired simultaneously at the end of fermentation by maintaining a constant temperature of 26 °C, this low temperature restricted the rapid growth of cells in the prior period of fermentation and reduced HSAF productivity throughout the fermentation process. Consequently, it was necessary to develop a proper temperature supply strategy to ensure efficient fermentation to achieve a high concentration, high yield, and high productivity of HSAF. Thus, a novel two-stage temperature control strategy was proposed and demonstrated to not only improve the production of HSAF but also considerably increase HSAF productivity and yield. This result can be explained by the following two aspects. Increasing the culture temperature at the beginning of fermentation allows cells to grow stronger and faster by consuming substrates, thereby shortening the fermentation period. On the other hand, improvement in substrate uptake could provide more precursors and energy for cell growth and product synthesis, which is consistent with other studies [25]. Moreover, energy consumption can be reduced during the early stage of fermentation due to the elevated temperature, which will be beneficial for the industrialized production of HSAF in the future.

Temperature is one of the important environmental factors affecting microbial growth and metabolite synthesis, as any enzymatic reaction in biochemistry is related to temperature [26]. However, the temperatures required for microbial growth and product formation differ in most cases [14]. A phased temperature control strategy can provide ideal temperatures for both cell growth and product synthesis and is widely used in generating fermentation products such as α-cyclodextrin glucosyltransferase [27], 1,3-propanediol [14], and glutathione [28].

## Conclusions

In this study, various culture conditions affecting the production of HSAF were systematically optimized, and it was found that high temperature during the early stage of fermentation favoured the rapid growth of cells, while low temperature was best for HSAF production in the late stage of fermentation. A two-stage temperature control strategy was proposed and demonstrated to be a better strategy for HSAF production, productivity, and yield than a single temperature-controlled process. This efficient method is very promising for the large-scale industrial production of HSAF.

## Additional file

Additional file 1: **Table S1.** Experimental design and responses of PBD. **Table S2.** Experimental design and responses of BBD. **Table S3.** Regression analysis of BBD. **Figure S1.** Response surface curves showing the interface of the medium capacity, culture temperature, and fermentation time. (DOCX 667 kb)

## Abbreviations

ANOVA: Analysis of variance
BBD: Box-Behnken design
DCW: Dry cell weight
DHA: Docosahexaenoic acid
HPLC: High-performance liquid chromatography
HSAF: Heat-stable antifungal factor
NRPS: Nonribosomal peptide synthetase
PBD: Plackett-Burman design
PKS: Polyketide synthase
PTMs: Polycyclic tetramate macrolactams
RSM: Response surface methodology
