# Supplementary material for: Efficient production of heat-stable antifungal factor through integrating statistical optimization with a two-stage temperature control strategy in Lysobacter enzymogenes OH11
Source: BMC Biotechnol. 2018 Oct 24;18:69. doi: 10.1186/s12896-018-0478-2 (PMC6201579; doi:10.1186/s12896-018-0478-2)
Supplement: Supplementary file 1 — Table S1. Experimental design and responses of PBD. Table S2. Experimental design and responses of BBD. Table S3. Regression analysis of BBD. Figure S1. Response surface curves showing the interface of the medium capacity, culture temperature, and fermentation time. (DOCX 667 kb) [file 12896_2018_478_MOESM1_ESM.docx]

**
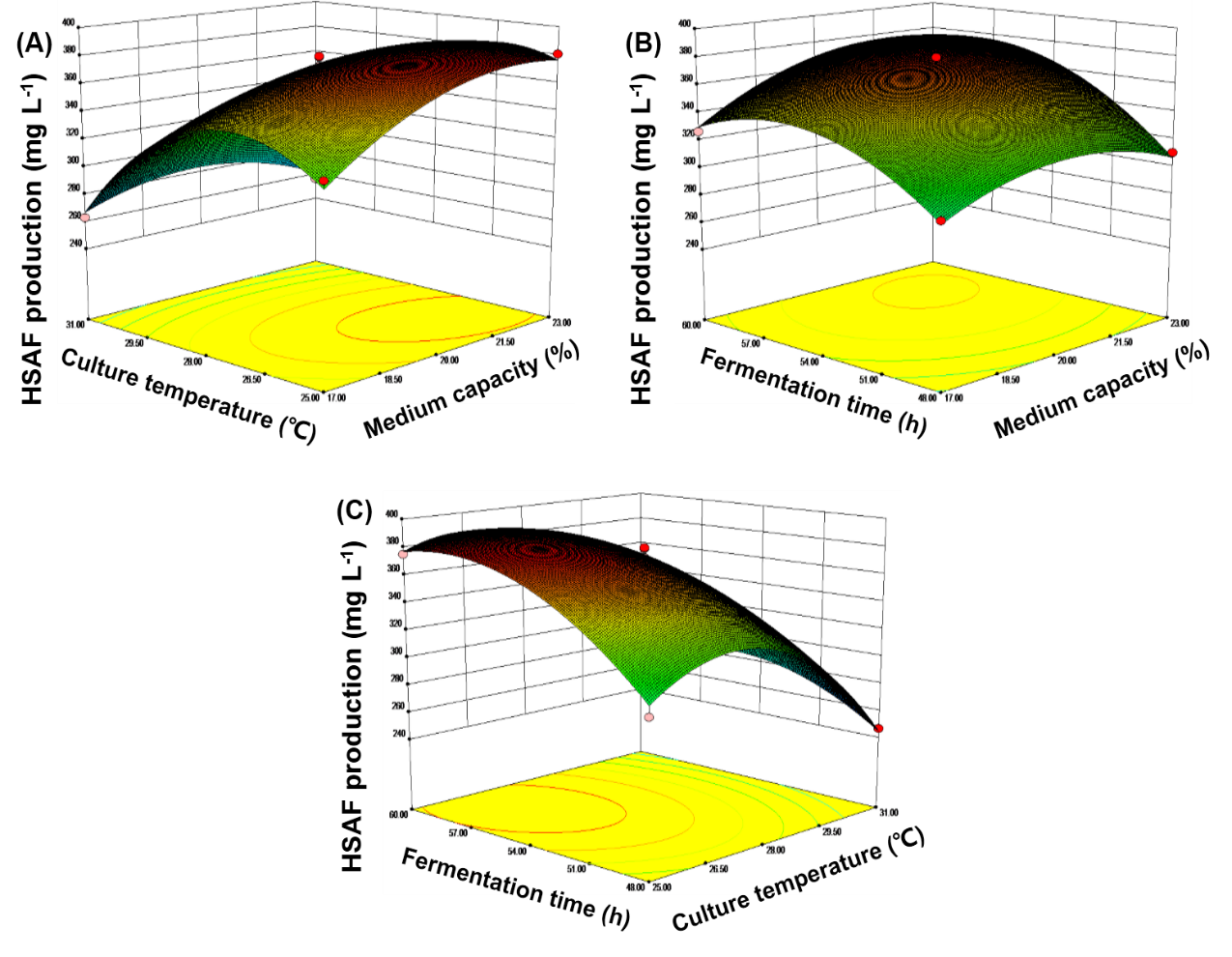
**

**Fig. S1**

**Table S1** Experimental design and responses of PBD

| Run | Inoculation amount  X_1_(%) | Medium capacity  X_2_(%) | Initial pH  X_3_ | Culture temperature  X_4_ (°C) | Rotation speed  X_5_ (r min^-1^) | Fermentation  time  X_6_ (h) | HSAF production (mg L^-1^) |
| --- | --- | --- | --- | --- | --- | --- | --- |
| 1 | -1 | +1 | +1 | +1 | -1 | -1 | 283.76 ± 15.13 |
| 2 | +1 | +1 | -1 | -1 | -1 | +1 | 277.52 ± 13.42 |
| 3 | +1 | +1 | +1 | -1 | -1 | -1 | 218.36 ± 14.92 |
| 4 | +1 | -1 | +1 | +1 | +1 | -1 | 240.00 ± 14.41 |
| 5 | -1 | +1 | +1 | -1 | +1 | +1 | 314.00 ± 15.12 |
| 6 | -1 | -1 | -1 | +1 | +1 | +1 | 325.30 ± 14.08 |
| 7 | -1 | -1 | -1 | -1 | -1 | -1 | 182.32 ± 13.45 |
| 8 | +1 | +1 | -1 | +1 | +1 | +1 | 328.16 ± 14.11 |
| 9 | +1 | -1 | +1 | +1 | -1 | +1 | 347.28 ± 14.09 |
| 10 | +1 | -1 | -1 | -1 | +1 | -1 | 193.76 ± 13.44 |
| 11 | -1 | +1 | -1 | +1 | +1 | -1 | 293.6 ± 15.24 |
| 12 | -1 | -1 | +1 | -1 | +1 | +1 | 256.48 ± 14.55 |

**Table S2** Experimental design and responses of the BBD

| Assay | Coded values | | | Actual values | | | Response |
| --- | --- | --- | --- | --- | --- | --- | --- |
|  | X_2_ | X_4_ | X_6_ | Medium capacity (%) | Temperature  (°C) | Fermentation  time (h) | HSAF production (mg L^-1^) |
| 1 | +1 | -1 | 0 | 17 | 25 | 54 | 324.84 ± 15.25 |
| 2 | +1 | +1 | 0 | 23 | 31 | 54 | 255.95 ± 14.32 |
| 3 | -1 | 0 | -1 | 17 | 28 | 48 | 299.79± 16.16 |
| 4 | 0 | +1 | -1 | 20 | 31 | 48 | 246.46 ± 14.86 |
| 5 | 0 | 0 | 0 | 20 | 28 | 54 | 372.22 ± 14.44 |
| 6 | 0 | 0 | 0 | 20 | 28 | 54 | 378.65 ± 12.51 |
| 7 | 0 | -1 | -1 | 20 | 25 | 60 | 374.92 ± 14.24 |
| 8 | 0 | 0 | 0 | 20 | 28 | 54 | 371.77 ± 14.68 |
| 9 | 0 | 0 | 0 | 20 | 28 | 54 | 379.35 ± 15.43 |
| 10 | 0 | +1 | +1 | 20 | 31 | 60 | 275.65 ± 13.72 |
| 11 | +1 | -1 | 0 | 23 | 25 | 54 | 380.45 ± 14.05 |
| 12 | +1 | 0 | +1 | 23 | 28 | 60 | 369.54 ± 15.16 |
| 13 | 0 | -1 | -1 | 20 | 25 | 48 | 295.53 ± 14.66 |
| 14 | -1 | +1 | 0 | 17 | 31 | 54 | 262.93 ± 14.90 |
| 15 | -1 | 0 | +1 | 17 | 28 | 60 | 326.45 ± 13.55 |
| 16 | 0 | 0 | 0 | 20 | 28 | 54 | 370.68 ± 15.06 |
| 17 | +1 | 0 | -1 | 23 | 28 | 48 | 310.21 ± 13.35 |

**Table S3** Regression analysis of BBD

| Origin source | SS^a^ | DF^b^ | MS^c^ | F-Value | Probe＞F | Significance |
| --- | --- | --- | --- | --- | --- | --- |
| Mode | 38228.66 | 9 | 4247.63 | 94.12 | < 0.0001 | ** |
| X_2_ | 1304.07 | 1 | 1304.07 | 28.90 | 0.0010 | ** |
| X_4_ | 14007.20 | 1 | 14007.20 | 310.37 | < 0.0001 | ** |
| X_6_ | 4732.19 | 1 | 4732.19 | 104.86 | < 0.0001 | ** |
| X_2_X_4_ | 979.38 | 1 | 979.38 | 21.70 | 0.0023 | ** |
| X_2_X_6_ | 266.83 | 1 | 266.83 | 5.91 | 0.0453 | * |
| X_4_X_6_ | 630.01 | 1 | 630.01 | 13.96 | 0.0073 | ** |
| X_2_^2^ | 1695.51 | 1 | 1695.51 | 37.57 | 0.0005 | ** |
| X_4_^2^ | 9873.40 | 1 | 9873.40 | 218.77 | < 0.0001 | ** |
| X_6_^2^ | 3293.86 | 1 | 3293.86 | 72.99 | < 0.0001 | ** |
| Residual error | 315.91 | 7 | 45.13 |  |  |  |
| Lack of fit | 247.93 | 3 | 82.64 | 4.86 | 0.0803 |  |
| Pure error | 67.98 | 4 | 17.00 |  |  |  |
| Sum | 38544.57 | 16 |  |  |  |  |
| R^2^=0.9918 R^2^(Adj)=0.9813 | | | | | | |
| ^a^ Sum of Squares; ^b^ Degree of freedom; ^c^ Mean Square; *indicate significant (<0.05); ** indicate highly significant (<0.01). | | | | | | |
